# Gravitational Influence on Human Living Systems and the Evolution of Species on Earth

**DOI:** 10.3390/molecules26092784

**Published:** 2021-05-08

**Authors:** Konstantinos Adamopoulos, Dimitrios Koutsouris, Apostolos Zaravinos, George I. Lambrou

**Affiliations:** 1 School of Electrical and Computer Engineering, Biomedical Engineering Laboratory, National Technical University of Athens, Heroon Polytechneiou 9, Zografou, 15780 Athens, Greece; jkadamopoulos18@gmail.com (K.A.); dkoutsou@biomed.ntua.gr (D.K.); 2 Department of Life Sciences, School of Sciences, European University Cyprus, 1516 Nicosia, Cyprus; 3 First Department of Pediatrics, Choremeio Research Laboratory, National and Kapodistrian University of Athens, Thivon & Levadeias 8, Goudi, 11527 Athens, Greece

**Keywords:** evolution, microgravity, hypergravity, astrobiology, gravitational biology

## Abstract

Gravity constituted the only constant environmental parameter, during the evolutionary period of living matter on Earth. However, whether gravity has affected the evolution of species, and its impact is still ongoing. The topic has not been investigated in depth, as this would require frequent and long-term experimentations in space or an environment of altered gravity. In addition, each organism should be studied throughout numerous generations to determine the profound biological changes in evolution. Here, we review the significant abnormalities presented in the cardiovascular, immune, vestibular and musculoskeletal systems, due to altered gravity conditions. We also review the impact that gravity played in the anatomy of snakes and amphibians, during their evolution. Overall, it appears that gravity does not only curve the space–time continuum but the biological continuum, as well.

## 1. Introduction

The question of how gravity affected the evolution of species is a topic of intense research during the last years, especially after the increased interest towards space/planetary colonialization. The understanding of how species evolved under the gravitational influence is of paramount importance in order to understand how life could emerge in other planetary systems.

The first molecule of life was the RNA, which probably constituted the intermediate precursor between DNA and proteins. This is also supported from our current knowledge on the catalytic role of RNA apart from the carrier of genomic information. As an example, it has been known that the Formose, or Butlerov reaction is a possible prebiotic reaction for the synthesis of sugars, as for instance ribose, necessary for RNA formation [1]. Yet, the Formose reaction would have produced a large amount of molecules, probably most of them useless for the prebiotic process. Another interesting problem arising for the origin of life was that actin, for instance, was not known to be present at the early events, yet it appeared that the polymerization of glycine along with smaller molecules led to the formation of actin and therefore the cytoskeleton [2]. Another major obstacle that the creation of life had to surpass was the concept of chirality. In chemical synthesis, a reaction produces equal amounts of L- and D- products, yet in life, reactions take place with the appropriate portions of L- and D-chiral substances, as is the case of amino acids and sugars. Thus, how is life aware of the necessary proportions of L-, D-chiral substances and the appropriate combinations in order for life itself to emerge?

To this problematic several interesting hypotheses have been stated, where we could highlight a recent postulation relating to this “confinement”. As the early planet-forming events created a number of solid and semi-solid structures, which changed numerous times due to terraforming events, provided the necessary platform for the first chemical reactions. One of the first questions on the origin of life was the existence of N_2_ and CH_4_ in the first atmosphere, with hypotheses stating that N_2_ and CO_2_ were initially favored, followed by CH_4_ after the emergence of methanogenic bacteria [2]. The presence of “reaction-friendly” surfaces formed by minerals and other solid surfaces along with life-supporting gases presented the first “furnace” for the formation of life molecules. Another significant aspect that was stated was the parameter of “confinement” [1,2]. This term describes the realization of chemical reactions in the confined space of solid surfaces, which could eventually lead to the formation of life-appropriate molecules [1].

During the life-forming procedure, all parameters can be held variable, yet one physical quantity was constant, and that was gravity, which is considered to have remained unchanged during the last four billion years [3]. On Earth, life was able to form and evolution led to the development of living matter. Despite all terra-forming events, gravity was the constant factor. Therefore, it is plausible to assume that gravity has been a major force in shaping life in our planet [4].

In the present study, we reviewed the effects of altered gravitation in different physiological systems, focusing on the cardiovascular, immune, vestibular and musculoskeletal systems. We primarily discuss experiments that have been conducted in either actual or simulated conditions of altered gravity, aiming to determine the affected functionalities at the gene, cellular or organ levels. We also discuss the topic of gravitational sensing by cellular organisms and how this is related to their function and evolution. As it is difficult to define the gravity-dependent biological changes within one generation, only noticeable alterations can be studied during the course of a single generation. Nevertheless, gravity seems to have driven the evolution of species living in different gravitational environments on Earth.

### 1.1. Using Gravitational Force (g) an Experimental Variable

Gravity decreases with distance, as the force between two different masses is reversely proportional to the square of the distance described as Equation (1):

(1) $$F=G\frac{m_{1}m_{2}}{R^{2}}$$

where *m*_1_ and *m*_2_ are the two masses, *R* is the radius between them and *G* is the gravitational constant. Thus, it is possible for a spacecraft to reach a sufficient distance from Earth, where an individual or object, inside the vessel, would “feel” very little of the gravitational force (also termed as microgravity). Nevertheless, this is not why things “float” on a vessel in orbit. Particularly, the International Space Station (ISS) orbits our planet at an altitude between 320 and 400 km. Earth’s gravity is approximately 90% of its surface at that altitude. Gravity causes all objects to fall with the same velocity in vacuum conditions (i.e., without frictional forces). This speed is irrespective to the objects’ mass, as it is only dependent upon the height of fall and the gravitational acceleration (*g*), which is formally denoted as Equation (2):

(2) $$u=\sqrt{2gh}$$

where *u* is the speed an object develops just before it touches the ground, *g* is the gravitational acceleration and *h* is the height of the fall.

**Figure 1:**
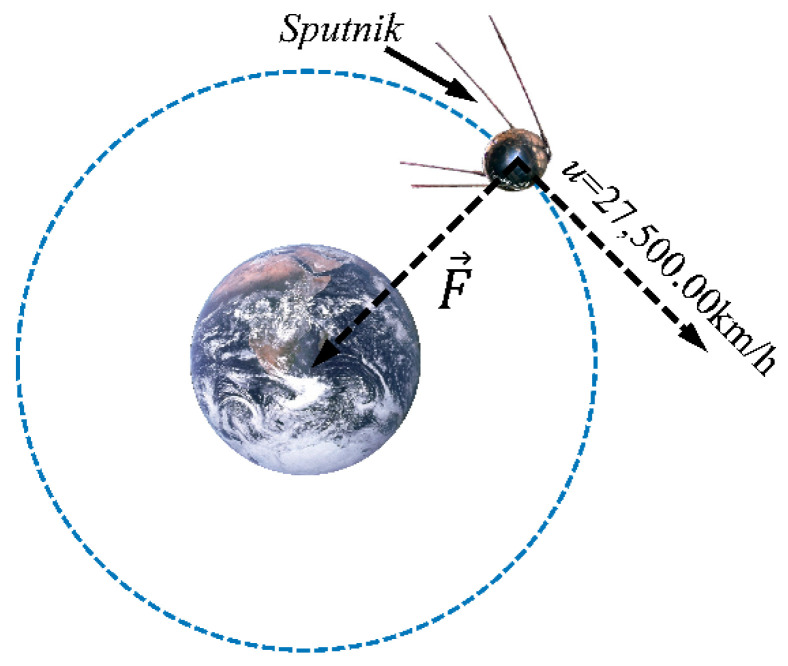
An orbiting object around Earth (Sputnik), with its velocity and applied forces.

An orbiting spacecraft moves at 27,500 km per hour, and the orbit of its “fall” follows the orbit and gravitational force of the Earth (Figure 1). Due to this fact, a spacecraft keeps falling toward the ground and never “hits” it. Thus, every spacecraft that is in a circular orbit above Earth is actually in a free fall around the planet [5].

The procedures for real microgravity experimentation are expensive and scarcely available. Therefore, a variety of platforms have been developed in order to simulate altered gravity conditions that are comparable to the real microgravity conditions. Different ground-based facilities, such as random positioning machines, clinostats and others, have been constructed due to the aforementioned reason.

The continuum of gravitational force could be divided into special domains, in which experiments must use different technologies and equipment. In particular, the microgravity domain of 0 < *g* < 1, extended experiments require both a spacecraft and a centrifuge (actual). Either horizontal (near zero *g*) or tilted clinostat on Earth is needed in order to simulate this microgravity domain. In the domain > 1 *g* a centrifuge is essential; this experimental setup can take place either on Earth or in space. Nominally 0 *g* is attainable only in space for long periods and the 1 *g* condition is easily attained on Earth, as it is the condition under which terrestrial life forms have evolved. Simulations can be used as a guide to what is happening under, real, reduced gravity conditions [6,7,8,9].

The knowledge on the effects of microgravity is derived from experimental models both in vivo and in vitro. Experiments have been performed in space, in cells in culture, animals (e.g., mice) [10,11], plants (e.g., maize) [12], bacteria and nematodes [13], but most importantly, in humans [14,15,16]. A significant source of information comes from the health monitoring of astronauts in space and the physical examination of astronauts upon their landing. Astronauts are exposed to various health risks, due to the long-term presence under microgravity conditions and the exposure to cosmic rays. Therefore, their monitoring and health assessment is imperative. Astronaut monitoring includes the use of traditional methodologies, such as hematological and biochemical measurements, but also advanced techniques, such as biosensors [17].

Space monitoring has been developed through the years, with the first physical examinations including the evaluation of height and body mass, the measurement of numbers of breath, pulse rate, blood pressure, hematological parameters such as lymphocyte count, erythrocyte count and platelet count and biochemical measurements, which included electrolyte estimation, urine analysis, hematouria, proteinuria, glucosuria and finally stool examination, which included the microbiome examination of astronauts [18]. Yet, most importantly a significant examination included the examination of bone density and the determination of radioactive isotopes in the human body [18].

Since those initial evaluations, scientific progress has led to the advancement of physical examination in spaceflight. An interesting example was the NASA “Twins Study” [19]. This study was based on the very genuine idea, to investigate the effects of spaceflight in two identical twins, where the first is bound to Earth and the second remains in space for one year. Yet, the idea moved further on by using all available biomedical technologies, which included simple hematological and biochemical analysis, transcriptomic analysis, epigenomic, immunophenotype, metabolomics, microbiomics, proteomics, physiology and telomeric analysis [19]. The study showed that Earth-bound physiology and spaceflight physiology differ, and it has been apparent that microgravity affects the magnitude of the human physiology and not only isolated physiological systems.

### 1.2. Literature Mining

The search engines PubMed, ScienceDirect and Google Scholar were used with the keywords “Evolutionary biology, gravity” (104 results on PubMed sorted by best match), “Gravity and evolution” (1089 PubMed results), “Gravitational biology” (2625 PubMed results sorted by best match concerning all species, 808 results related to *Homo sapiens* and 10,076 results in Science Direct), “Universal Darwinism” (8533 results in Science Direct) and “Astrobiology and evolution” (19,700 results on Google Scholar). All references that arose from the identified articles were searched for relevant data. The end date of the literature search was set to 2020. Furthermore, PubMed database was used typing the keywords “musculoskeletal, gravity” (110 results), “vestibular, gravity” (206 results), “immune, gravity” (78 results), “cardiovascular, gravity” (276 results) and “microgravity, evolution” (filtered “other species”; 28 results). The end date of the literature search was set to 2020. The thorough search was focused on the most subjectively relevant articles.

## 2. Effects of Altered Gravity on *Homo sapiens* and Other Vertebrate Species

In general, humans adapt appropriately to the space environment and the conditions are not life threatening for at least a one-year stay in space [20]. However, some of the most common problems that appear after landing is dizziness and muscle weakness. Therefore, appropriate countermeasures, such as training of high-intensity and short duration, have to be developed in order to effectively minimize the aforementioned impairments [4,21]. Below, we reviewed the effect of gravity on various systems of the human body.

### 2.1. Gravity and the Thyroid Gland

Human’s cardiovascular, immune, vestibular and musculoskeletal systems present severe abnormalities in the altered gravity condition. Albi et al. (2017) supported that microgravity induces morphological and functional alterations within the thyroid gland [22]. Its physiological function is required for physical and mental health, as cardiovascular, musculoskeletal, nervous and immune systems are controlled by it. In particular, the authors treated FRTL-5 cells with the thyroid stimulating hormone (THS) at the onset of microgravity and fixed them just at the end of the microgravity period [23]. They also observed cytoskeletal changes of human FTC-133 cells rapidly after entrance into microgravity. Follicular cells are responsible for the production and the secretion of the thyroid hormones thyroxine (T4) and triiodothyronine (T3) and constitute the mayor cell type in the thyroid gland. On the other hand, experiments with normal human primary thyroid follicular epithelial cells (Nthy-3-1-ori) at hypergravity conditions (1.8 *g*) showed that the expression of Integrin Subunit Alpha 10 (ITGA10) does not depend on the gravitation environment [22,23,24].

### 2.2. Gravity and the Cardiovascular System

Post-flight orthostatic intolerance, cardiac atrophy and heart rhythm disturbances are some of the indications proving that microgravity affects the human cardiovascular system [25]. NASA demonstrated that mean arterial pressure is reduced in the case of long-term exposure to microgravity [19]. Is the evolution of mammalian blood pressure affected by gravity? Generally, the total height of the blood column above the heart increases with respect to the size of the body, and the central systemic arterial blood pressure is positively related to it. Thus, the hearts of larger animals should pump harder against gravity and the body’s higher peripheral resistance [26]. The hydrostatic pressure at the bottom of a column of fluid is calculated as the product of fluid density, gravitational acceleration and the vertical height of the column [26]. Analyzing the diastolic, systolic and mean blood pressure in 47 mammalian species, and using nonlinear analyses, White et al. (2014) showed that the mean blood pressure differs significantly from a 10 g mouse to a 4 t elephant [26]. In addition, blood pressure did not differ significantly, from the predicted one, based on the vertical distance between the head and heart, indicating that the pressure that is needed to perfuse the capillaries at the top of the body may be less among larger species [26].

Fuentes et al. (2015) investigated the reaction of progenitors, isolated from the neonatal and adult human heart, in a microgravity environment by quantifying alterations in functional parameters, gene expression and protein levels after 6 days of 2D clinorotation [25]. This study showed that age might play a significant factor relevant to the effects of the exposure of cardiovascular progenitors to conditions of simulated microgravity. Neonatal progenitors seemed to acquire characteristics of dedifferentiating cells; whereas the expression of markers for endothelial and cardiomyogenic differentiation was higher in adult cardiac progenitors [25].

Jha et al. (2016) engineered microscale progenitor cardiac spheres from human pluripotent stem cells, and exposed them to simulated microgravity using a random positioning machine for 3 days, during the phase of their differentiation to cardiomyocytes. Highly enriched cardiomyocytes with high viability (90%) occurred from the aforementioned process. Increased proliferation and viability of cardiac progenitors, and upregulation of genes associated with survival at the early stage of differentiation, were observed in the 3D culture under microgravity conditions [27].

Wnorowski et al. (2019) utilized human induced pluripotent stem cell derived cardiomyocytes (hiPSC-CMs) to study the effects of microgravity on cell-level cardiac functionality and gene expression. The cells were cultured aboard the ISS for 5.5 weeks, and alterations in calcium handling were observed. Almost 2700 genes were differentially expressed among flight, post flight and ground control samples [28].

### 2.3. Gravity and the Immune System

Immune cells are also sensitive to altered gravity conditions. Bucheim et al. (2019) stated that a specific group of stressors on humans, which are able to provoke an aberrant immune activation, triggered a sustained release of endocannabinoids in a long duration spaceflight [29].

Bonyaratanakornkit et al. (2005) evaluated the differential transcriptional response of primary human T cells’ genes in simulated freefall using the random positioning machine, and showed that gravity affects signaling pathways that could cause the increased susceptibility to infection [30]. The authors noticed 99 significantly upregulated genes during early T cell activation in normal gravity. Their work suggests that gravity constitutes a key regulator of immune response and that its absence either impedes or fully prevents signaling pathways that are essential for the early activation of T cells [30].

Chang et al. (2012) examined the supposition that microgravity-exposed T cells inhibit the transcription of immediate early genes. T cells were stimulated on board (ISS) with anti-CD28 and concanavalin A (ConA). Simulation of 1 *g* simultaneous control was created by an on-board centrifuge in order to isolate the effects of gravity from other spaceflight variables. The results showed that activated T cells in the *g*- and 1 *g*- environment, exhibited differential gene expression patterns. In particular, 47 genes were significantly downregulated in the *g* condition, as the microarray expression analysis demonstrated after 1.5 h of activation. The transactivation of cAMP Responsive Element Modulator (CREM), Rel/NF-B and SRF targets was reduced and the expression of REL gene targets was considerably inhibited. Furthermore, gene connectivity analysis showed that gravity conditions during the spaceflight might lead to ineffective proinflammatory host defenses against infectious pathogens due to inhibition of the TNF pathway. The aforementioned results indicate that gravity is responsible for inhibiting the transactivation of key immediate early genes [31].

Girardi et al. (2014) used simulated microgravity via a ground-based rotating wall vessel bioreactor in order to analyze the expression profiles of miRNAs and mRNAs in human peripheral blood lymphocytes (PBL) [32]. Forty two miRNAs, among which miR-9-3p, miR-9-5p, miR-150-3p, miR-155-5p and miR-378-3p, were differentially expressed compared with 1 *g* of incubated PBLs. A correlation of the aforementioned miRNAs with *IFNG, IL17F, PDCD4, PTEN, NKX3-1, GADD45A* and other functionally similar genes was identified. These genes are involved in the immune response, apoptosis and cell proliferation. In particular, the classification of the correlated genes evidenced significant enrichment in the inflammatory response, signal transduction, regulation of programmed cell death, cell proliferation and response to stress [32]. In a recent report Chowdhury et al. also stated that microgravity induced differential expression in 370 transcripts related to the oxidative stress response, carbohydrate metabolism and regulation of transcription [33].

On the other hand, Thiel et al. (2017) simulate their gravitational environment through a combination of parabolic flight with suborbital ballistic rocket, 2D clinostat and centrifuge experiments. They studied the stability of non-activated human Jurkat T lymphocytic cells’ gene expression. Their experiments showed that 97–90% of all transcripts were not significantly altered in the microgravity environment where strict controls were used for excluding all possible factors of influence. Almost one third of the transcripts (20–40%) remained unchanged between 10^−4^ and 10^−2^ *g* and 10–20% of them remained totally unaltered in any gravitational conditions, i.e., between 10^−4^ and 9 *g*. Thus, the referred study is an indicator that gene expression is highly stable in reduced gravitational conditions [34].

Similarly, experimental models have been used in order to address the question of microgravity effects on blood monocytes. Human studies have highlighted that astronauts, manifest an increase in monocytes, neutrophils and T-helper cells but a decrease in natural killer cells [35]. Previous studies have indicated the changes in monocyte physiology during spaceflight, where it has been found that monocytes lose their ability to trap bacteria after 5 days under microgravity [36]. The effect of immunosuppression with respect to monocytes was confirmed by a later study, where it was found that circulating monocytes lost their ability to invoke an immune response after a parabolic flight, with alternating gravitational forces [37]. While monocytes presented activation markers under hypergravity and normal gravity it manifested immunosuppression-related surface markers [37]. These findings were confirmed by a recent study, where it was found that the inflammatory potential of monocytes was reduced through inhibition of the JNK pathway and the p38/MAPK pathway [38]. Further on, in vitro studies have been performed in microgravitational conditions, where it has been found that J111 cells manifested cytoskeletal changes in F-actin, β-tubulin and vinculin, whereas F-actin was reduced under microgravity [39].

Finally, studies with autophagy inhibitors have shown that it is also affected by microgravity. Isoluquiritigenine (ISL), administered to RAW264.7 cells, a macrophage/monocyte-like cell line, inhibited cell differentiation to osteoclasts through RANKL by inhibiting autophagy, as evidenced by the reduction of TRAP-positive multinucleated osteoclasts, F-L3-ring formation and Bec-, which are markers of autophagy, while modifying the pattern of intracellular distribution of Beclin1 and LC3II. ISL caused a decrease in the protein levels of Atg5-Atg12, Beclin 1 and the LC3II/LC3I quotient and in a dose-dependent manner in RAW264.7 cells [40].

As in the case of monocytes, neutrophils have also been the topic of study under microgravity conditions. In a previous study it has been reported that the neutrophils manifested increased post-flight levels as compared to preflight levels [41]. While, the monocytes manifested immunosuppressive properties due to microgravity, neutrophils did not manifest any changes in the cell’s ability to engulf bacteria, yet no changes in neutrophils’ granularity or surface markers were observed [41]. These findings were confirmed by a recent report, which have demonstrated that in astronauts’ neutrophils remained at the same levels in preflight, flight and postflight specimens [42]. As in previous cell lineages, B-lymphocytes manifested a decrease in a mouse model under simulated microgravity [43]. Additionally, as in the previous reports on cytoskeletal effects of microgravity, B-lymphocytes also manifested cytoskeletal changes, which were directly linked to the microgravitational influence [43]. Further on, in a murine model, which remained for one month in space, a 41% decrease in the B-lymphocyte population was observed in the murine spleen, one week after landing [44]. In a recent study, it was reported that, besides bone mass alterations, decreased B-cell lymphopoiesis was also observed. In particular, in a mouse model under hind limb unloading no change was observed in the hematopoietic stem cell population and in multipotent precursor cells. Yet, a significant decrease in B-progenitor cells was apparent from day three and throughout the duration of the experiment [45]. Noteworthy, B-cell population reduction was stress- and inflammation-independent, indicating a secondary mechanism of action. The mechanism through which B-cell poiesis is restricted has been found to be probably via the decreased expression of EBF and PAX5 and STAT5-mediated IL7 signaling [45]. Another study has shown that antiorthostatic suspension reversed the B-cell to T-cell ratio in mice spleen and at the same time it was able to differentially affect the cell’s mitogenic responses. In agreement with the study of Lescale et al. (2015) those changes were stress- and inflammation-independent, also suggesting a secondary mode of action in lymphocyte population physiology [46]. In the same study, it was revealed that B-cells are more prone to changes after simulated microgravity as compared to T-cells, whereas among the T-cell population T-helper cells are more sensitive than cytotoxic T-cells [46].

Concerning the effects of microgravity on immune cells, all studies agree that microgravity has two main effects; first it reduces the population size of immune cells and also reduces their immunoresponsive potential, and second it enhances cytoskeletal changes, which are directly linked to the effect of microgravity. These changes are probable reasons for the increased vulnerability to pathogens in the astronaut population [45,46].

### 2.4. Gravity and the Vestibular System

Our guidance system—the vestibular—is able to control posture, stability of the body, the activity of the sympathetic nerve, arterial pressure, feeding behavior, muscle and bone metabolism, eye movements and vertical orientation with respect to gravity. This system contains otolith organs and semicircular canals that sense linear and angular acceleration, respectively. The vestibular system is highly plastic and appears to be affected upon exposure to altered gravitational circumstances [47].

Jamon et al. (2014) showed that peripheral sensory organ adjusts the mass of otoconia and the innervation of the sensory epithelium so as to adapt to the level of applied gravitational levels [48]. Hallgren et al. (2016) studied and compared the pre- and postflight ocular counter-rolling response (OCR), a reflex produced by the activation of the gravity sensors in the inner ear that stabilizes gaze and posture during head tilt, in a group of 25 astronauts, and found a dramatic decrease of the OCR response upon their return, whilst the otolith-mediated response was back at preflight levels 9 days after their return [49].

Furthermore, Reschke et al. (2018) compared the OCR from six astronauts before, during and after a 4–6 day spaceflight with the OCR measures acquired before and after a spaceflight that lasted 4–9 months [50]. As far as short-duration spaceflights is concerned, the response returned to normal within 2 h and no OCR as noted during head tilt in microgravity; whereas the amplitude of OCR was reduced, without any changes in the asymmetry of OCR between the right and left head tilt, for several days after their return to Earth. Their data indicate that otolith-mediated reflexes adapt to microgravity through a long-lasting process [50].

### 2.5. Gravity and the Musculoskeletal System

Some of the most striking effects of microgravity include the musculoskeletal system. Up-to-date experience includes the study of muscle and skeleton in space stations, which concerned a time duration of almost a year. In previous studies it has been reported that during International Space Station (ISS) missions, astronauts experienced a reduction in bone mineral density (BMD) by 2.5–10.6% in the lumbar vertebrae, decrease of femur BMD by 3–10%, and some manifested 1.7–10.5% decrease in BMD of the femoral neck [51]. From the first space flights it has become apparent that astronauts experienced a 1–6% decrease in the spine, femoral neck, trochanter and pelvis per month, which is however varying between individuals [51].

Muscles and bones related to posture and weight weaken without gravitational load. One major kind of damage observed in spaceflight is bone loss. There is an imbalance between bone formation and resorption according to several studies and spaceflight missions [52]. Cultured muscle fibers in microgravity were reported to be atrophied by 10–20%, compared to ground controls due to a decrease in protein synthesis [3]. The concentration of bone resorption markers was reported to be increased on the other hand, even if good nutrition and physical training was implemented during the spaceflight [53].

Myoblasts, being an inherent component of the musculoskeletal system, are also affected by hypergravity. A study performed in mice myoblasts concluded that there is increased myosin expression and subsequently a myoblast differentiation rate in 20 *g* [54]. Ikawa et al. (2011) also showed increased bone mineral density in rat trabecular bone under hypergravity (3 *g*), via the reduction in both bone resorption and formation, as indicated by biochemical and histomorphometric analysis [55]. Moreover, gravity has been shown to hugely affect the cell cytoskeleton. This disruption of the normal cell architecture could affect a plethora of procedures ranging from cell signaling to cell proliferation and apoptosis [56,57].

Kacena et al. (2002) studying osteoblasts submitted in a 1–4 *g* gravitational field, noted an increase in the number and thickness of actin filaments, fibronectin and vinculin, but no change in the proliferation rate [58]. In a similar study, an increase in actin fiber density, but not in number, was pointed even in low *g* forces [59]. The results of both studies are in accordance with even more recent research performed on human tendon cells, cultured in 15–20 *g* for 16 h [60].

Bradamante et al. (2018) studied osteogenic stem cell differentiation in microgravity in order to discover how human bone marrow stem cells (hBMSCs) react to a two week exposure in ISS when treated with the osteo-inducer 1,25-dihydroxy vitamin D. Their results provide evidence of cell cycle arrest, although without any indications of adipogenesis, senescence and apoptosis. Thus, hBMSCs seem to revert to a quiescent state as they are influenced by the microgravity environment. This condition could be reversible because of the upregulation of exosomal miRNAs [61]. Similar results were provided in experiments in mice being exposed in microgravity [62]. In addition, cell cycle arrest was also observed in another study; however, without any evidence for normal terminal differentiation markers [63].

## 3. Gravity and Evolution

The four principal forces are the nuclear strong forces, nuclear weak forces, the electromagnetic forces and gravitational force. The gravitational force is constant and it can be assumed that it has been affecting life for the last four billion years. Recent experimental results have shown that complexity in organisms is directly related to the necessary environment for sustaining life, i.e., their existence. In other words, microbiota can survive outside a spaceship with minimal protection, while mammals need complex sustaining environments (i.e., spaceships) for supporting life [4].

As life emerged in water, the initial effect of gravity was annihilated by buoyancy, yet the organisms required a specialized mechanism for enduring water pressure. As species moved to land, they were challenged by the gravitational force and they had to create new mechanisms, i.e., musculature, for locomotion. The common denominator in all species was that they had to develop different mechanics from one stage of evolution to the next.

Gravity seems to have an evolutionary role in snakes. This is evident by the difference in the position and structure of their internal organs, which is attributed to their evolution and adaptation in different gravitational environments. In particular, snakes that crawl up and down trees are constantly coping with gravity. On the other hand, sea snakes spend their life swimming and neutrally float, whilst land snakes move in a horizontal level. The orientation of each snake species to the direction of the gravitational force is different, according to its environment. Lillywhite H.B. (1988) first noticed that a tree snake’s heart was closest to the brain and supported that blood should not be transferred distantly from the heart to the brain [64]. Thus, he suggested that sea snakes are mainly influenced by gravity, considering that they faint with increased gravity, whilst tree snakes are gravity-tolerant.

Perez et al. (2019) examined the position of specific internal organs in 72 snakes across 13 species. The results corroborated the influence of gravity on the morphology of the cardiopulmonary system, and indicated that the gravity-sensitive vascular lung varied the most among all organs [65].

Wright and Turko (2016) attempted to determine whether the plasticity of extant amphibious fishes could indicate the strategies used during the evolution of terrestriality in tetrapods. The researchers observed a reversible plasticity in locomotor function in the mangrove rivulus *Kryptolebias marmoratus* [66]. Brunt et al. (2016) supported that the terrestrial locomotor performance of *Kryptolebias marmoratus* was improved, even in the absence of exercise training, due to reversible alterations to its oxidative skeletal muscle [67]. Their experiments showed that air-exposed fishes demonstrated improved locomotor performance (i.e., they jumped further and for longer) compared to their counterparts held in water. Physical changes, such as hypertrophy and angiogenesis in the oxidative muscle, were reversed within two weeks of returning to water. The exact stimulus for the aforementioned alterations remains unknown, however it is likely to be partly related to the increase of the gravitational level [67].

Load bearing structures are also influenced by gravitational conditions. Some support structures, such as limps, become less functioning in microgravity. Thus, the physiology of human legs over time without gravity constitutes a problem that has to be dealt with for long-time spaceflights. On the other hand, the microbes are trying to avoid their exposure to solar radiation, using gravitational force as an environmental signal when they migrate. Microbes could suffer most from radiation in the case of a gravitational absence, which should lead to an evolutionary selection of the species bearing radiation resistance [4].

**Figure 2:**
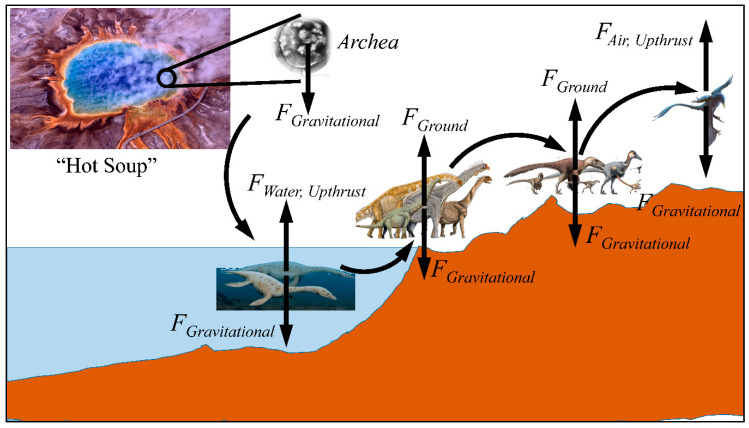
A diagrammatic representation of the evolution on Earth and the force of gravity acting on all stages of evolving life. As organisms evolved from the primary microorganisms to the formation of life in water, the transition to land and then air was constantly under the effect of gravitational forces (**Legend**: the images of the “hot soup”, archaea and dinosaurs were obtained from https://en.wikipedia.org/wiki/Dinosaur, under the CC BY 2.0 license, Accessed on 3 October 2020).

The exact mechanism of gravity sensing is still unknown. It is possible that even in the primeval conditions of life on Earth, the first microorganisms were formed under the influence of gravity (Figure 2). One of the early works on the subject presented a possible explanation for the forces that shaped life [68]. In particular, this hypothesis postulated that the effects of life forming events took place in a specific order that forces acted on the first cells in a specific order. In a simplified form the life-forming forces appear in a hierarchy, which can be described as Equation (3) [68]:

(3) $$F=Ca^{N}$$

where, *F* is the force under examination, *C* is the size of the organism under evolution, *a* is the radius between the force generator and the organism under evolution and *N* is the power of the size of the organism. If Equation (3) is log-transformed, it is derived (Equation (4)) [68]:

(4) logF=logC+Nloga

Equation (4) is a linear function with slope *N* and intersect log *C*. This report has shown that as the size of the organism increases, i.e., the weight, thus gravity, is the principal force, while when the size decreases electrical forces are in primary play [68].

In another report, another aspect is considered, the role of water. Under the influence of gravity, water behaves as a liquid with specific viscosity. In space, i.e., under microgravity, water properties change and thus its diffusion properties [69]. This is fundamental since diffusion was considered one of the principal physical phenomena in evolution [70]. The role of water becomes more apparent in the case of plants, which are able to withstand gravitational forces without a support mechanism. Plant growth and water transport (for example in trees that exceed 10 m) takes place through capillary phenomena, defying gravity. The same mechanism takes place in space, as plants were able to grow and reproduce in space [4,71].

## 4. Discussion

Gravity constitutes the only constant environmental parameter, during the period of evolution of living matter on our planet. From the first time-points of the emergence of life, to the evolution of the first aquatic species, the constantly evolving species experienced a gravitational load. Early terrestrial species experienced an increase in the magnitude of the gravity’s force vector, which probably changed their orientation related to the gravity vector and increased in height. As a result, they were forced to evolve adaptive mechanisms to move fluids and structures against the gravitational forces or for directional changes. Land species increased in complexity and size, and as a result, they required the appropriate supportive structures, with respect to their load. For example, crawling species do not require the same mechanisms to counter the effect of gravity, but they need mechanisms to overcome the increased friction forces. This happened because they were alternating between horizontal and vertical positions. Species that stood on extremities, developed musculature and bone structures to support their bodies against gravitation. On the other hand, birds faced lift and drag obstacles related both to gravity and air density before they were capable of flying, thus, they evolved a musculoskeletal system along with lighter bone structures that could provide adequate thrust [4].

The biological role of gravity was questioned three decades ago, when Alpatov, Antipov and Tairbekov posed the question of whether any of the processes that exist in a cell is gravity-dependent and the likelihood of cell adaptation to weightlessness [72]. In 2003, Morey-Holton, from NASA Ames Research Center, reviewed relevant studies to spaceflight and ground-based experiments and concluded that “gravity shapes life” [4]. Gravity has remained constant throughout the entire history of Earth. Therefore, we can assume that its influence was also constant throughout the emergence and evolution of life upon Earth. Today there is evidence that our musculoskeletal [73,74,75], cardiovascular [76,77,78] and immune systems [79,80,81] function under the tight control of gravity. 

The biology and growth of plants, also seems to be influenced by the gravitational force [7,79,82,83,84]. Nevertheless, the role of gravity on our body’s systems and the evolution of living systems on Earth have not been adequately addressed.

## 5. Conclusions

The complexity of organisms is directly related to the complexity of the sustaining environment. Previous studies have shown that there are significant differences between species living in conditions on the Earth’s surface and species under microgravity. Especially in humans, those changes include various physiological systems, as for example, immunity, locomotion and metabolism. Yet, one of the main characteristics we would highlight is the role of musculature and skeletal physiology. The fact that humans lose a significant amount of bone mass in space probably restricts the choices of space exploration for future colonialization. That means the planets of choice should probably be those with conditions similar to those on the Earth, especially with respect to the gravitational force. In this sense, Venus is the closest planet that resembles the Earth with respect to gravity.

Gravity seems to have affected the ongoing evolution of species. A more in-depth investigation to his would require frequent and long-term experimentations in space conditions or environments of altered gravity. Each organism should be studied throughout numerous generations in order to determine the profound biological changes in their evolution. Ultimately, considering the aforementioned studies, it appears that gravity does not only curve the space–time continuum, but the biological continuum, as well.

## Data Availability

Not Applicable.
